# Effect of ER:YAG Laser Irradiation on the Load-Bearing Capacity and Reliability of All-Ceramic Zirconia Crowns

**DOI:** 10.3390/ma19153196

**Published:** 2026-07-27

**Authors:** Natália Piffer Pasquali, Patrícia Moreira de Freitas, Márcia Borba, Paula Benetti

**Affiliations:** 1 Post-Graduation Program in Dentistry, University of Passo Fundo (UPF), Passo Fundo 99052-900, RS, Brazil; natipasquali@gmail.com; 2 Special Laboratory of Lasers in Dentistry (LELO), School of Dentistry–USP, São Paulo 05508-000, SP, Brazil; pfreitas@usp.br; 3 Division of Dentistry, School of Medical Science, The University of Manchester, Manchester M13 9PL, UK; marcia.borba@manchester.ac.uk

**Keywords:** ceramics, mechanical testing, dental porcelain, compression fracture

## Abstract

**Highlights:**

- Er:YAG irradiation did not impair the zirconia crown’s fracture strength and reliability.
- Monolithic zirconia crowns had higher load-bearing capacity than veneered ones.
- Veneered zirconia showed chipping and delamination, while monolithic crowns failed catastrophically.

**Abstract:**

Background: This study evaluated the effect of Erbium:Yttrium–Aluminum–Garnet (Er:YAG) laser irradiation on the fracture load, failure mode, and reliability of monolithic and porcelain-veneered zirconia crowns. Methods: Monolithic ultra-translucent zirconia crowns (5YZ-M, n = 30) and porcelain-veneered zirconia crowns (3YZ-V, n = 30) were divided into control (Ctrl-no surface treatment) and Er:YAG laser irradiation (Las) groups (n = 15). Las was performed on the cementation surface (250 mJ, 10 Hz, 2.5 W for 30 s). Crowns were adhesively cemented onto dentin-analog abutments and subjected to compressive load-to-failure testing. Data were analyzed by two-way ANOVA, Tukey’s post hoc test, and Weibull analysis (α = 0.05). Fractographic analysis was performed. Results: Laser irradiation did not affect the fracture load (*p* = 0.183). Monolithic crowns (5YZ-M) showed significantly higher fracture load than veneered crowns (3YZ-V) (*p* < 0.001). There was no effect of the interaction between factors (*p* = 0.150). The m-values were similar among groups, while the L_0_ of 3YZ-V-Ctrl was significantly lower than that of 5YZ-M. Veneered restorations predominantly showed chipping and delamination; whereas, monolithic crowns mainly exhibited catastrophic failures. Fracture origins were mostly located at the occlusal loading area. Conclusions: Er:YAG laser irradiation of the cementation surface did not affect the load-bearing capacity or structural reliability of zirconia crowns.

## 1. Introduction

Polycrystalline zirconia is widely used in contemporary prosthodontics due to its favorable combination of high fracture strength and toughness, and increasingly improved esthetics [1,2,3,4,5]. However, the long-term clinical behavior of zirconia-based restorations is influenced by multiple factors, including the ceramic microstructure and composition, surface treatment protocols, restoration design, veneering techniques, properties of the veneering ceramics, cementation strategies, and clinical handling procedures [5,6,7,8,9].

Despite its good mechanical properties, reliable bonding to zirconia remains a clinical challenge [8,10,11,12]. Zirconia is a polycrystalline and chemically inert ceramic, being resistant to conventional acid etching methods that are effective for feldspathic and glass-ceramics [8,10,11,12]. The bonding protocol most widely applied involves airborne-particle abrasion with alumina particles, with or without silica coating, followed by the application of resin cements containing functional phosphate monomers, such as 10-methacryloyloxydecyl dihydrogen phosphate (MDP) [10,11,12,13]. For 3 mol% yttria-stabilized tetragonal zirconia polycrystals (3Y-TZP), this approach has been shown to enhance micromechanical interlocking, chemical adhesion, and, in some cases, flexural strength through a transformation-toughening mechanism associated with the tetragonal-to-monoclinic phase transformation [9,11,14,15].

However, the clinical use of 3Y-TZP is limited by its relatively high opacity, resulting from the birefringence of tetragonal grains, which restricts its indication to posterior monolithic restorations or porcelain-veneered zirconia infrastructures [1,2,4,16]. Nevertheless, in bilayer restorations, the mismatch between the high-strength 3Y-TZP infrastructure and the weaker veneering porcelain or glass-ceramic, combined with restoration geometry, thickness variations, and thermally induced residual stresses, has been associated with chipping and delamination of the veneering layer [5,17,18,19].

To overcome esthetic limitations, high-translucency zirconia with increased cubic-phase content (approximately 50% in content) and higher yttria concentrations (4–5 mol%; 4Y-PSZ and 5Y-PSZ) have been introduced, enabling the fabrication of monolithic restorations in esthetically demanding areas [2,3,5,6,7,16,20,21,22]. Although these materials offer improved optical properties, their associated microstructural changes lead to reduced transformation-toughening capability and lower tolerance to surface damage [2,15,20,21,22]. Consequently, conventional airborne-particle abrasion may induce surface flaws and microcracks on the cementation surface, potentially compromising the mechanical reliability of these translucent cubic-containing zirconia restorations [10,14,23,24,25].

Therefore, alternative surface treatment strategies for translucent zirconia have been explored. Approaches such as airborne abrasion with particles of lower elastic modulus (e.g., silica-based particles) and laser irradiation, particularly when combined with resin cements containing chemically active agents like MDP, have shown promising results in terms of surface modification and bonding effectiveness [13,26,27,28,29,30,31]. Laser irradiation has been investigated as an alternative surface treatment to increase the surface energy of zirconia and enhance adhesion to resin cements, while potentially reducing the risk of microcracks and structural damage commonly associated with conventional airborne-particle abrasion [27,28,29,30,31,32,33,34,35,36,37,38,39,40]. Erbium:Yttrium–Aluminum–Garnet (Er:YAG) laser irradiation promotes surface modification through an ablation mechanism involving micro-explosions and evaporation of granules, increasing surface roughness, which may improve micromechanical retention and adhesion to zirconia surfaces [27,29,32,33,34,35,36,37,38,39,40]. Nevertheless, the effect of laser irradiation on the load-bearing capacity and structural integrity of zirconia crowns—especially high-translucency monolithic restorations—remains unclear and insufficiently documented in the literature [3,8,17,31].

The aim of the present study was to evaluate the effect of Er:YAG laser irradiation on the fracture load, failure mode, and reliability of monolithic and porcelain-veneered zirconia crowns. The first null hypothesis is that Er:YAG laser irradiation does not affect the fracture load and reliability of zirconia crowns. The second null hypothesis is that monolithic and veneered zirconia crowns exhibit similar fracture load and reliability.

## 2. Materials and Methods

The materials used in the study are described in Table 1.

### 2.1. Specimen Preparation

Cylinders (Ø20 × 120 mm) of a dentin-analog glass fiber-reinforced epoxy resin (G10) were milled to obtain simplified preparations of maxillary second premolars (N = 60). The preparations presented a 1.2 mm cervical chamfer and rounded angles between axial and occlusal walls [41].

All crowns were fabricated using a computer-aided design and computer-aided manufacturing system (CAD–CAM; InLab MC X5, Dentsply Sirona, Charlotte, CA, USA). Ultra-translucent zirconia (5Y-PSZ) crowns were designed as monolithic restorations using InLab 19 software (Dentsply Sirona), reproducing the anatomy of a maxillary second premolar. The axial and occlusal thicknesses were standardized at 2.0 mm and 3.0 mm, respectively, including cusps and primary grooves.

Conventional zirconia (3Y-TZP) was milled with an infrastructure design with axial and occlusal thicknesses of 1.0 mm and 2.0 mm, respectively. These infrastructures were veneered by a single trained operator using the conventional layering technique with feldspathic porcelain (Vita VM9, Vita Zahnfabrik, Bad Säckingen, Germany). Porcelain powder and modeling liquid were mixed at a 2:1 ratio and applied into a sectioned condensation silicone mold (Zermack, Badia Polesine, Italy) obtained from the external anatomy of the monolithic crowns, ensuring standardized contours. Three porcelain firing cycles were performed to achieve the final crown anatomy (total axial and occlusal thickness of 2.0 mm and 3.0 mm, respectively).

Sintering was performed as recommended by the manufacturer, in a Ceramill Therm 3 (Amann Girrbach, Mäder, Austria) furnace using a conventional cycle of 2 h holding time at 1450 °C, in a total cycle of approximately 8 h. After sintering, the crowns were cleaned in a sonic bath (Ultrasound Tubes Instrument; Cristofoli Ltda, Paraná, Brazil) and immersed in distilled water for 5 min to remove surface debris.

### 2.2. Surface Treatment

**Figure 1:**
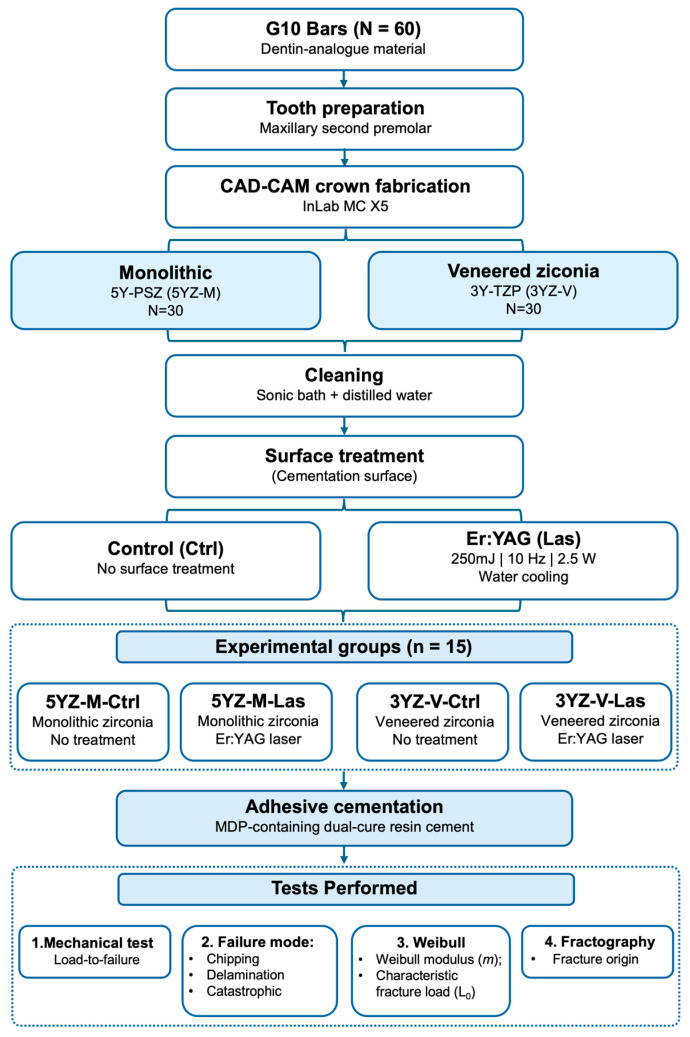
Flowchart of the experimental groups.

Monolithic and zirconia-veneered crowns were sub-divided according to the cementation surface treatment: control or laser-irradiated (Figure 1).

Er:YAG laser (LiteTouch^TM^, Light Instruments, Yokneam, Israel) of 2940 nm wavelength, R02-C handpiece and a AS7077X optical fiber (cylindrical sapphire, 0.8 mm diameter × 8 mm length; beam area of 0.005 cm^2^) was used under water irrigation (20 mL/min). Er:YAG laser irradiation of the internal (cementation) surface of the crowns was performed by a single trained operator. Laser settings were determined by a pilot study evaluating four different irradiation protocols for 3Y-TZP and 5Y-PSZ zirconia, varying pulse energy (200 or 250 mJ), repetition rate (10 or 15 Hz), power output (2.5 or 3.0 W), and irradiation angle (45° or 90°). Surface morphology was analyzed by optical microscopy and scanning electron microscopy (SEM), searching for irregularities, melting areas, and crack formation. All protocols produced only slight surface alterations, without visible defects. Laser irradiation at 250 mJ, 10 Hz, 2.5 W was selected for the present study (Table 2), because it promoted homogeneous surface modification while minimizing the risk of thermally induced damage associated with higher repetition rates.

### 2.3. Cementation Protocol

Surface conditioning of the G10 dentin-analog abutment was performed following the protocol described by Mosele et al. [24]. The procedure consisted of etching the cementation surface of the G10 dentin-analog abutments with 10% hydrofluoric acid (Condac; FGM, Joinville, Brazil) for 60 s, followed by rinsing with water and air-drying. A silane coupling agent (Ultradent, Indaiatuba, Brazil) was applied to the etched surface for 60 s. Subsequently, the adhesive system from Panavia F 2.0 resin cement (Kuraray, Tokyo, Japan) was applied for 20 s and light-cured for 10 s according to the manufacturer’s instructions.

Prior to cementation, the intaglio surface of the crowns was cleaned with air/water spray and dried. All crowns were adhesively cemented using a dual-cure resin cement (Panavia F 2.0; Kuraray, São Paulo, Brazil). Base and catalyst pastes were mixed in equal proportions and applied to the internal surface of each crown. The crowns were placed onto the corresponding abutment and maintained under a constant axial load of 750 g, that was applied to the crown occlusal surface. Excess cement was removed with a brush before polymerization, and chemical curing was allowed for 5 min. Light activation was performed for 40 s on each axial surface using a light-emitting diode unit (3200 mW/cm^2^ max intensity, VALO; Ultradent Products, Inc., Indaiatuba, Brazil), followed by additional light curing for 40 s on the occlusal surface. After cementation, specimens were stored in distilled water at 37 °C for up to 72 h prior to mechanical testing.

### 2.4. Fracture Load Test and Failure Mode Analysis

Fracture load testing was performed using a universal testing machine (DL 2000; EMIC, São José dos Pinhais, Brazil). A spherical stainless-steel indenter was positioned on the occlusal surface of each crown, contacting three points (a cusp-to-fossa relationship), with a polyester strip interposed to allow a more uniform stress distribution. A compressive load was applied at a crosshead speed of 0.5 mm/min until catastrophic failure. Specimens remained immersed in distilled water at 37 °C during the test. The maximum fracture load was recorded in Newtons (N).

Failure modes were analyzed according to fractographic principles using a light microscope (ZTX ZOM; Ningbo Wason Optical Instruments, Ningbo, China) at 80× magnification. Failures were classified as chipping (fracture of the ceramic without exposure of the infrastructure or abutment), delamination (fracture of the veneering ceramic with exposure of the infrastructure but not the abutment), or catastrophic failure (complete fracture of the crown with exposure of the abutment). Representative specimens from each failure mode were further examined by scanning electron microscopy (SEM) to identify fracture origins using fractography principles.

### 2.5. Statistical Analysis

Based on the observed effect size for the main factor restoration type (partial η^2^ = 0.213; Cohen’s f = 0.520), a minimum sample size of 45 specimens is required to achieve 80% power at α = 0.05 in a balanced 2 × 2 factorial design. Therefore, the sample size used in the present study (N = 60; n = 15 per group) is adequate to detect the main effect of restoration type.

Fracture load data passed the Shapiro–Wilk normality and the equal variance tests (*p* > 0.05). Fracture load data were analyzed by two-way ANOVA (factor 1: surface treatment, factor 2: type of restoration) and Tukey’s post hoc test (α = 0.05) (SigmaPlot v12; Systat Software, San Jose, CA, USA). Weibull analysis was performed to determine the Weibull modulus (m) and characteristic fracture load (L_0_). The 95% confidence intervals (95% CI) for the Weibull parameters were calculated using the Likelihood Ratio method (Weibull++, Reliasoft).

## 3. Results

No significant interaction was found between restoration type and surface treatment for fracture load (Table 3). Consequently, the main effects were evaluated independently. Surface treatment did not significantly affect fracture load; whereas, restoration type had a significant effect. Specifically, 5YZ-M crowns showed significantly higher fracture load values than 3YZ-V crowns, irrespective of surface treatment (Table 4).

**Figure 2:**
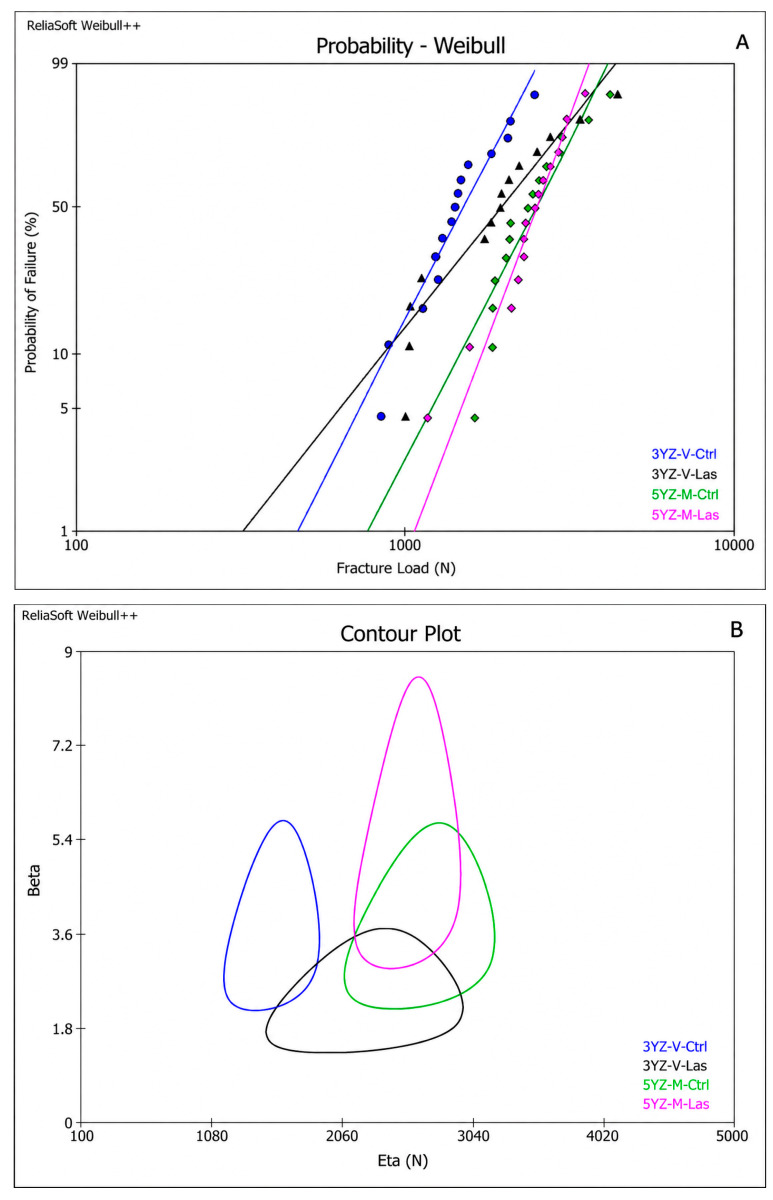
(**A**) Weibull graph showing the fracture load data for the experimental groups. (**B**) Contour plot showing the 95% confidence intervals for the Weibull modulus (beta) and the characteristic fracture load (Eta).

The Weibull analysis results are described in Table 5 and Figure 2. The Weibull modulus (m) was similar among the groups. The L_0_ of the 3YZ-V-Ctrl group was significantly lower than that of the 5Y-M-Ctrl and 5Y-M-Las groups as the 95% CI did not overlap.

**Figure 3:**
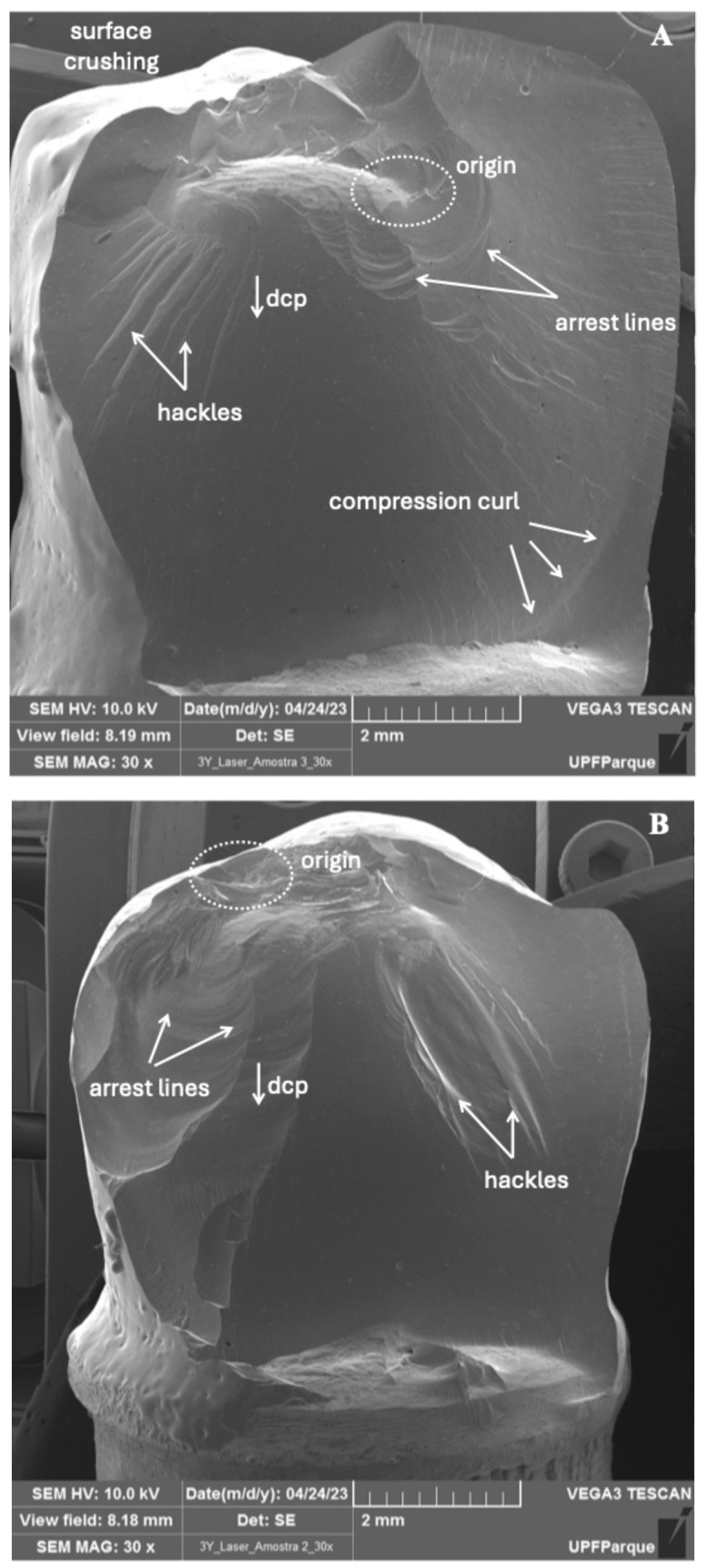

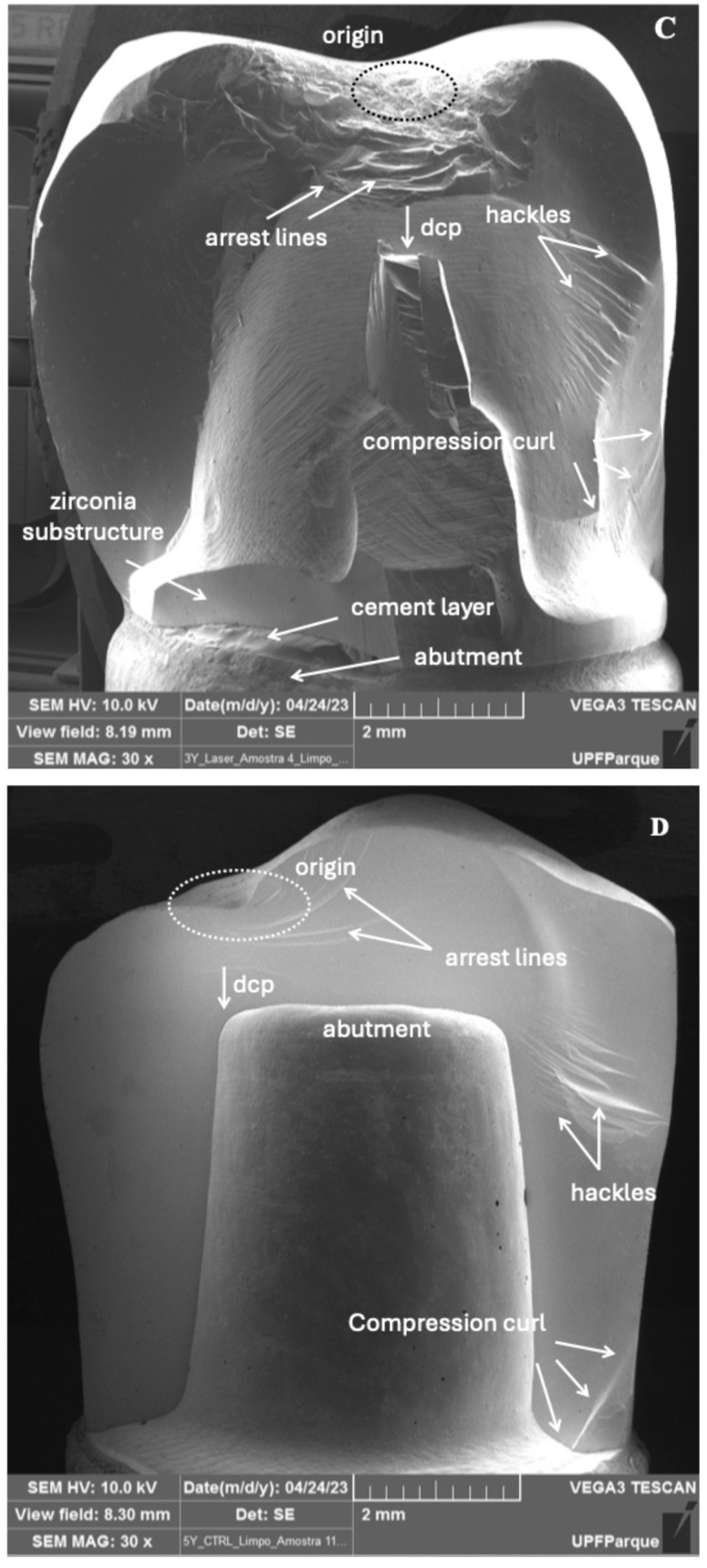
Representative SEM images of the failure modes. (**A**) delamination; (**B**) chipping; and (**C**) catastrophic failure of the veneered-zirconia specimens. (**D**) catastrophic failure of monolithic specimens. In catastrophic and chipping fractures, fractographic features such as hackle lines indicate crack propagation from the occlusal surface, near the contact area with the loading piston toward the cervical region. Delamination failures initiated below the loading area, with crack propagation along the zirconia–veneering ceramic interface, showing occlusal surface crushing and partial delamination of the veneering ceramic. Arrest lines were observed, suggesting crack deflection at the ceramics interface. Direction of crack propagation (dcp) is shown by the white arrow.

The results of the failure mode analysis are summarized in Table 6. Representative fracture modes were examined by SEM and are shown in Figure 3. In veneered crowns, chipping and delamination were the most frequent failure modes (Figure 3A,B); whereas, monolithic crowns predominantly exhibited catastrophic failures (Figure 3D). The origin of the fractures occurred mainly on the occlusal surface near the contact area with the loading piston (Figure 3B–D), and, in veneered crowns, at the zirconia–veneering ceramic interface (Figure 3A).

## 4. Discussion

This study investigated how Er:YAG laser treatment of the cementation surface affects the mechanical behavior of monolithic translucent zirconia and porcelain-veneered zirconia crowns. Based on our findings, the first null hypothesis that Er:YAG laser irradiation does not affect the fracture load and reliability of zirconia crowns was accepted. Yet, the second null hypothesis was partially accepted as the type of restoration did not affect the reliability but influenced the fracture load values.

In the present study, laser irradiation was investigated as an alternative surface treatment for bonding zirconia. An Er:YAG laser with a wavelength of 2780 nm was applied using controlled parameters (250 mJ pulse energy, 10 Hz, 2.5 W, for 30 s, 2 mm distance, and incidence angles of 45° and 90°). Under these conditions, laser irradiation did not significantly affect the fracture load of either monolithic or veneered zirconia crowns, suggesting that the protocol used was not able to introduce new defects to the materials or compromise their mechanical behavior. These findings are consistent with previous studies demonstrating that low to moderate Er:YAG energy settings can modify zirconia surfaces without inducing structural damage [32,34,35,36,37,38,39].

Cavalcanti et al. [42] reported that high-energy Er:YAG irradiation (600 mJ) induced superficial fusion, mass loss, and deep cracks in 3Y-TZP zirconia; whereas, lower energy levels (200–400 mJ) produced surface roughness patterns comparable to airborne-particle abrasion. Similarly, previous investigations [30,32] have suggested that laser irradiation may represent a viable alternative to alumina airborne-particle abrasion when performed with controlled parameters, including pulse energy and irradiation duration, and when excessive roughness capable of inducing critical surface defects is avoided. In agreement with these observations, the present results demonstrate that the chosen protocol of Er:YAG laser irradiation does not compromise the mechanical behavior of cemented zirconia crowns.

These findings further support previous studies [27,28,29,37,38] that investigated surface treatments developed to improve zirconia bonding to resin cement, which have emphasized the potential of laser-based approaches as alternatives to airborne-particle abrasion. In particular, Tzanakakis et al. [38,39] demonstrated that femtosecond laser irradiation enhanced bond strength without inducing the undesirable surface damage commonly associated with conventional mechanical abrasion, thereby supporting the rationale for laser treatment of zirconia surfaces.

Regarding restoration design, monolithic 5YZ crowns exhibited significantly higher fracture load values than porcelain-veneered 3YZ. This result is consistent with previous investigations reporting superior load-bearing capacity of monolithic zirconia compared to veneered-zirconia systems [5,6,7,17,18,19]. However, as different types of zirconia were used, the fracture load values cannot be attributed exclusively to the restoration design. Other variables such as the zirconia composition, the absence of veneering ceramic, restoration geometry, thickness distribution, and residual thermal stresses generated during porcelain firing may all have contributed to the mechanical behavior. Consequently, the present findings should be interpreted as reflecting the overall performance of the tested restorative systems. Nevertheless, the absence of the veneer–core interface in monolithic restorations likely eliminates an important site for stress concentration, crack initiation, and failure that has been consistently identified in bilayer restorations [5,17,18,19].

Failure mode analysis further supports these findings. Monolithic crowns exhibited predominantly catastrophic failures; whereas, veneered restorations showed a higher prevalence of chipping and delamination. This difference may be attributed to the mismatch in mechanical properties between zirconia and veneering ceramic, residual thermal stresses generated during porcelain firing, and variations in restoration geometry and ceramic thickness [5,17,18,19,24]. The absence of veneering ceramic in monolithic crowns increases the amount of energy required to induce minor fractures, such as chipping, which is recognized in the literature as a major advantage of monolithic zirconia restorations [5,6,7,17,18,19,42]. Fractographic analysis revealed that fracture origins were predominantly located on the occlusal surface, particularly at the contact area with the loading piston, indicating that the loading configuration generated higher stress concentration in this region than at the cementation surface. In veneered-zirconia restorations, crack propagation frequently occurred along the zirconia–veneering ceramic interface.

Although laser irradiation did not significantly affect overall fracture load, the veneered zirconia group irradiated with Er:YAG (3YZ-V-Las) exhibited a characteristic fracture load (L_0_) comparable to that of the monolithic groups. Catastrophic failures of 3YZ-V-Las group were more frequent than the non-irradiated veneered zirconia (3YZ-V-Ctrl). Laser-induced modification of the cementation surface may have enhanced cement wetting and micromechanical interaction with the MDP-containing resin cement. The improvement of the intaglio surface quality can increase the functional stiffness of the crown–cement–abutment complex, allowing some specimens to withstand higher loads before failure [11,12,13,27,28,29,31]. Similar to van der Waals heterostructures, where interfacial defects alter electronic dynamics and tribological behavior, laser-induced modifications on the zirconia bonding surface may influence interfacial interactions and stress-transfer mechanisms across the cemented restorations [43]. Surface defects and topographical changes can affect the physicochemical characteristics of the interface, potentially modifying load distribution and energy dissipation during mechanical loading [43]. However, the findings of the present study indicate that the alterations induced by ER:YAG laser irradiation were not sufficient to compromise the overall mechanical integrity or load-bearing capacity of the cemented zirconia crowns. These observations reinforce the concept that the mechanical performance of complex systems is often governed by interfacial phenomena, even when the resulting outcomes differ, such as frictional behavior in two-dimensional materials and fracture resistance in zirconia-based restorations. As the load threshold increased, failures tended to shift from veneer-limited events (chipping) toward more extensive fractures involving the infrastructure and abutment exposure. In veneered zirconia restorations, where multiple competing failure sites coexist, including occlusal contact damage and the zirconia–veneer interface, small variations in residual thermal stresses, veneer thickness, and interfacial integrity may further increase data scatter and promote the transition from partial veneer fractures to catastrophic failures in a greater number of specimens.

The limitations of this study include the absence of artificial aging protocols, such as thermocycling and mechanical fatigue, which may have led to an overestimation of the long-term fracture load of the tested restorations. Under clinical conditions, zirconia crowns are subjected to cyclic masticatory loading, thermal variations, and a humid oral environment, all of which can promote slow crack growth, degradation of the cementation interface, and potential changes in mechanical reliability over time [2,3,8,20,21,22]. Therefore, the fracture load values reported in the present study represent the initial load-bearing capacity of the restorations rather than their long-term clinical performance.

In addition, low-temperature degradation (hydrothermal aging) of zirconia, which is particularly relevant for yttria-stabilized systems, was not evaluated and may affect surface properties and structural stability after prolonged exposure to oral conditions [2,15,20,21,22]. The use of a single resin cement and a standardized cementation protocol also limit the generalization of the findings, as different adhesive systems and surface treatment strategies may interact differently with laser-treated zirconia surfaces [8,11,12,13,25,27,28,29,30,31]. Finally, although a dentin-analog substrate was used to simulate clinical conditions, it cannot fully reproduce the complex biomechanical behavior of natural tooth structures and periodontal support. Fractographic analysis indicated that fracture origins were predominantly located on the occlusal surface of the crowns, particularly at the contact area with the loading piston. Therefore, the stress distribution induced by the loading method may not have sufficiently stressed the intaglio surface that was modified by the Er:YAG laser treatment. Nevertheless, not all crowns could be conclusively evaluated by fractographic analysis, meaning that fractures originating from the cementation surface cannot be completely excluded.

## 5. Conclusions

Er:YAG laser irradiation of the cementation surface does not affect the fracture load and the reliability of all-ceramic zirconia crowns. Monolithic translucent zirconia crowns exhibited superior load-bearing capacity compared with veneered zirconia restorations. These findings suggest that Er:YAG laser treatment may be considered a safe surface modification approach with respect to the mechanical performance of zirconia crowns under the conditions evaluated. Future studies should investigate the long-term effects of laser irradiation under fatigue loading and aging conditions to further establish its clinical applicability.

## Figures and Tables

**Table 1:** Information and clinical indication of the materials used in the study.

Commercial Name (Manufacturer)	Chemical Composition *	Description *
Ceramill ZI (3Y-TZP) (Amann Girrbach AG, Mäder, Austria)	ZrO_2_ + HfO_2_ + Y_2_O_3_: ≥99.0 Y_2_O_3_: 4.5–5.6 HfO_2_: ≤5 Al_2_O_3_: ≤0.5 Other oxides: ≤1	Slightly translucent zirconia. Flexural strength: 1200 ± 150 MPa Elastic modulus (E): ≥200 GPa Vickers hardness: 1300 ± 200 HV10 Coefficient of thermal expansion (CTE): 25–500 °C: 10.4 ± 0.5 10–6/K Chemical solubility: <100 μg/cm^2^
Ceramill Zolid FX-White (5Y-PSZ) (Amann Girrbach AG, Mäder, Austria)	ZrO_2_ + HfO_2_ + Y_2_O_3_: ≥99.0 Y_2_O_3_: 9.15–9.55 HfO_2_: ≤5 Al_2_O_3_: ≤0.5 Other oxides: ≤1	Ultra translucent zirconia Flexural strength: 700 ± 150 Mpa Elastic modulus (E): ≥200 GPa Vickers hardness: 1300 ± 200 HV10 CTE 25–500 °C: 10.1 ± 0.5 10–6/K Chemical solubility: <100 μg/cm^2^
Vita VM9—Base Dentin 3M3 (Vita Zahnfabrik, Bad Sackingen, Germany)	-	Fine-structured natural feldspathic veneering ceramic for zirconia dioxide infrastructure
NEMA G10 (G10) (International Paper)	Epoxy resin reinforced with glass fiber	Dentin analog
Panavia F 2.0 (Kuraray Noritake Dental Inc., Tokyo, Japan)	PANAVIA F 2.0 Paste (A Paste/B Paste) A Paste: 10-Methacryloyloxydecyl dihydrogen phosphate (MDP). Hydrophobic aromatic dimethacrylate. Hydrophobic aliphatic dimethacrylate. Hydrophilic aliphatic dimethacrylate. Silanized silica particle. Silanized colloidal silica. dl-Camphorquinone. Catalysts. Initiators. B Paste: Hydrophobic aromatic dimethacrylate. Hydrophobic aliphatic dimethacrylate. Hydrophilic aliphatic dimethacrylate. Silanized barium glass particle. Surface-treated sodium fluoride. Catalysts. Accelerators. Pigment.	Dual-cure resin cement (light-curable and/or self-curable), radiopaque, for ceramic, composite resin, and metal restorations

* Data provided by the manufacturer.

**Table 2:** Laser irradiation settings.

Description	Laser Er:YAG *
Crystal	Sapphire
Beam area	0.0028 cm^2^
Output power	2.5 W
Pulse energy	250 mJ
Repetition rate	10 Hz
Irradiation time	30 s
Irradiation distance	2 mm from the surface
Irradiation pattern	Scanning
Beam diameter	0.8 mm

* Based on Moretto et al. [29].

**Table 3:** Results of the two-way ANOVA for fracture load according to surface treatment and restoration type.

Source of Variation	df	SS	MS	F	*p*-Value
Surface treatment	1	753,265.1	753,265.1	1.814	0.183
Restoration type	1	6,292,334.3	6,292,334.3	15.152	<0.001 *
Surface treatment × Restoration type	1	882,479.4	882,479.4	2.125	0.150
Residual	56	23,255,414.3	415,275.3	—	—
Total	59	31,183,493.0	—	—	—

* Significant at α = 0.05.

**Table 4:** Maximum fracture load mean (standard deviation) and Tukey post hoc comparisons for the main effects.

Factor	Level	Mean (SD)	LS Mean (N)	Tukey *
Surface treatment	Control	2379.6 (654.1)	1934.5	A
	Laser	2371.8 (559.4)	2158.5	A
Restoration type	Monolithic	1489.3 (426.3)	2370.3	B
	Veneered	1956 (871.3)	1722.7	B

* Different letters indicate statistically significant differences according to Tukey’s test (α = 0.05).

**Table 5:** Mean fracture load (SD—standard deviation), characteristic fracture load (L_0_), Weibull modulus (m), and fracture load for a 5% failure probability (L_5%_), with respective 95% confidence intervals (95% CI) for the experimental groups.

Groups	L_0_ *	L_0_–95%CI	m *	m–95%CI	L_5%_	L_5_–95% CI
5YZ-M-Ctrl	2624.2 A	2249.9; 3034.4	3.8 A	2.5; 5.2	1202.6 AB	753.2; 1609.5
5YZ-M-Las	2563.8 A	2293.7; 2848.3	5.3 A	3.3; 7.5	1460.5 A	1013.8; 1812.6
3YZ-V-Ctrl	1645.6 B	1409.8; 1903.5	3.8 A	2.5; 5.3	753.1 B	465.3; 1012.0
3YZ-V-Las	2212.1 AB	1743.8; 2767.5	2.5 A	1.6; 3.4	664.5 AB	317.7; 1045.8

* Values followed by the same letter in the same column are similar according to the 95%CI analysis.

**Table 6:** Frequency of failure modes per experimental group.

Groups	Chipping % (n)	Delamination % (n)	Catastrophic % (n)
5YZ-M-Ctrl	7 (1)	0 (0)	93 (14)
5YZ-M-Las	20 (3)	0 (0)	80 (12)
3YZ-V-Ctrl	40 (6)	53 (8)	7 (1)
3YZ-V-Las	27 (4)	53 (8)	20 (3)

## Data Availability

The data presented in this study are available on request from the corresponding author. The data are not publicly available due to ongoing related research.

## Abbreviations

The following abbreviations are used in this manuscript:

3Y-TZP	3 mol% yttria-stabilized tetragonal zirconia polycrystal
4Y-PSZ	4 mol% yttria partially stabilized zirconia polycrystal
5Y-PSZ	5 mol% yttria partially stabilized zirconia polycrystal
3YZ-V	Porcelain-veneered 3Y-TZP zirconia crowns
5YZ-M	Monolithic 5Y-PSZ zirconia crowns
3YZ-V-Ctrl	Control group of porcelain-veneered 3Y-TZP zirconia crowns
3YZ-V-Las	Er laser-irradiated group of porcelain-veneered 3Y-TZP zirconia crowns
5YZ-M-Ctrl	Control group of monolithic 5Y-PSZ zirconia crowns
5YZ-M-Las	Er laser-irradiated group of monolithic 5Y-PSZ zirconia crowns
Al_2_O_3_	Aluminum oxide
ANOVA	Analysis of variance
CAD–CAM	Computer-aided design and computer-aided manufacturing
CI	Confidence interval
CTE	Coefficient of thermal expansion
Ctrl	Control
dl-Camphorquinone	dl-Camphorquinone photoinitiator
E	Elastic modulus
Er	Erbium
G10	Glass fiber-reinforced epoxy resin
HfO_2_	Hafnium oxide
Hz	Hertz
HV	Vickers hardness
Las	Laser irradiation
L_0_	Characteristic fracture load
L_5%_	Fracture load corresponding to 5% probability of failure
m	Weibull modulus
MDP	10-Methacryloyloxydecyldihydrogenphosphate
MPa	Megapascal
N	Newton
nm	Nanometer
PSZ	Partially stabilized zirconia
SD	Standard deviation
SEM	Scanning electron microscopy
W	Watt
Y_2_O_3_	Yttrium oxide
ZrO_2_	Zirconium dioxide
